# The Foreign Journals: Anatomy and Physiology

**Published:** 1841-04

**Authors:** 


### PART THIRD.

### Selections from the British and Foreign Journals.

### I. THE FOREIGN JOURNALS.

### ANATOMY AND PHYSIOLOGY.

*Observations and Experiments on the Functions of the Pneumogastric and Spinal Accessory Nerves.* By Professor ARNOLD, of Zurich.

THE following observations are abridged from an article in the Archives Générales de Médecine, founded on a work entitled "Bemerkungen über den Bau des Hirns und Rückenmarkes," published at Zurich in 1838, which we have not seen. Our space allows us to give little more than the general conclusions. Arnold's experiments seem to have been contemporary with Reid's and Valentin's, and fully confirm the views of the former so far as they extend, both in regard to the influence of the par vagum on respiration and digestion; but Arnold's analysis of the *mode* of influence is not nearly so complete as Reid's. They also fully coincide with Valentin's statements, as to the *solely sensory* character of the par vagum at the roots, and the acquisition of motor power by inosculation with the spinal accessory. To prevent confusion we adopt the language of the author, who in common with most Germans designates the pneumogastric nerves as the tenth pair, and the spinal accessory of Willis as the eleventh.

The experiments of Arnold were made with great care on fowls and pigeons, by simultaneously dividing the two pneumogastric nerves. We cannot detail these experiments, but group their results in the form of answers to the following questions:

I. *Is the pneumogastric a sensitive or a motor nerve?* Arnold shows that previous experiments cannot decide this question, because the pneumogastric nerve immediately after it passes from the cranium is united with the fibres of the internal branch of the thirteenth [eleventh?] pair. Therefore, on dividing it in the neck, a part of the spinal accessory is also cut, and the consequent phenomena result from the deranged functions of the two nerves. A division of the roots of the nerve within the cranium thus appears the only way of solving the question as to its functions.

The section of this nerve in the neck is not so painful as many physiologists have remarked. But the pain is very acute on dividing the superior laryngeal nerves. This explains the sensibility of the parts to which the pneumogastric is distributed, the mucous membrane of the glottis being far more sensitive than that of the œsophagus and stomach. The true explanation of this fact is found in the formation of the pneumogastric plexus, to which all the branches of the tenth part do not equally contribute. The superior laryngeal nerves have no extended connexion with the plexus gangliiformis, while that part which descends to the œsophagus and stomach principally contributes to form this plexus. Now as it is an *established physiological fact* [?] that the nerves which pass through plexuses conduct impressions to the brain much more slowly and less distinctly than other nerves, the varying sensibility of the stomach and glottis is explained in the distribution of the pneumogastric nerve. Arnold goes on to argue that this nerve is not a mixed, but a purely sensitive one, and on the following grounds: 1. It takes its origin from the posterior part of the medulla oblongata, like the greater portion of the fifth pair, and it has the same connexion with this part of the brain as the posterior roots of the spinal nerves have with the spinal marrow. 2. The disposition of the roots of the pneumogastric corresponds exactly to that of the spinal nerves, and with the larger portion of the fifth pair; that is to say, the roots remain isolated until they reach the ganglion which results from their union. The roots of the motor nerves, on the contrary, always reunite in the cranium or the vertebral canal into one or many trunks. This difference is very distinctly shown by comparing the larger portion of the fifth pair with its smaller portion, with the third, fourth, and sixth pairs, the tenth, eleventh, and twelfth, etc., etc. 3. The pneumogastric is provided with a ganglion, similar to the posterior roots of the spinal nerves and part of the trigeminal nerve, and this ganglion greatly resembles in its internal and external structure those of the spinal nerves. These combined arguments convince the author that the pneumogastric is a purely sensitive nerve.

II. *What influence has a division of the pneumogastric nerve over the general state of health?* General depression of the vital powers was observed in all Arnold's experiments, which he explains on the supposition that the division induces an incomplete transformation of venous into arterial blood. The influence of the operation on nutrition could not be determined as the animals died too quickly after the division. One dog became very lean, but the author ascribed this to the frequent vomitings, for when they ceased the dog recovered his condition. The nerve does not appear to exercise further influence on isolated organs, for the eyes of the fowls and pigeons preserved their natural state till near death.

III. *Is the sensation of hunger communicated by the tenth pair?* The experiments of M. Arnold convince him that the sensations of hunger and thirst depend on the integrity of these nerves. The voracity observed after their division is not opposed to this opinion: it proves on the contrary that the animals have not a distinct sensation of the state of their stomach. They devour food not so much to satisfy hunger as for the pleasure of eating and tasting it.

IV. *What influence does the pneumogastric nerve exercise over secretion, and the quantity and quality of the gastric juice?* Many physiologists, as Brodie, Wilson Philip, Tiedemann, etc., believe that the gastric juice ceases to be secreted after the division of this nerve. Others, as Ware, Finlay, Mayer, Brachet, etc., have seen the secretion of a juice less acid continued during some time. Arnold has never observed an absence of the gastric juice, and he does not believe that this nerve exercises a direct influence over either the quality or quantity of it.

V. *Are the contractions of the œsophagus and stomach under the influence of the pneumogastric nerve?* In answer to this question it would appear that the influence the division of this nerve has over these contractions is to be attributed to the internal branch of the spinal accessory nerve being also cut off.

VI. *What is the influence of the tenth pair over chymification?* In all the cases where Arnold divided these nerves, he found that the grains in the gizzard were entirely softened, and their internal parts altogether changed; and not only those grains in contact with the walls of the stomach, but also those in the midst. The grains were enveloped in a species of chyme, which fluid was also rejected by many fowls through the beak during life. The function of chymification then is not really changed, though it is deranged because the contractions of the stomach, or of the gizzard in fowls, are diminished. This result is directly opposed to the observations of Brodie, Wilson Philip, and others, who thought that the secretion of the gastric juice ceased after the section of the nerve, although the movements of the stomach continued.

VII. *What is the influence of the tenth pair on respiration and the respiratory movements?* Arnold found that in general the number of respiratory movements diminished after the section of these nerves. In some cases the diminution augmented in a uniform manner as death approached: in others the respirations diminished at once, but afterwards were feebly raised: in others again this elevation was followed by a new diminution. It does not appear that the respiratory movements are determined by the tenth pair, though this nerve exercises an indirect influence in communicating to the brain the sensations [impressions] produced in the lungs. The internal branch of the spinal accessory nerve, on the other hand, exercises a direct influence in determining the contractions of the muscles of the glottis.

VIII. *Is the tenth or the eleventh nerve the nerve of voice?* Rufus of Ephesus and Galen observed that the section of the tenth pair produced extinction of the voice, and Sœmmering called it the nerve of the voice. But Arnold is convinced that the vocal nerve is the eleventh, and not the tenth. At his instigation Bischoff made a number of researches in comparative anatomy to confirm this opinion. In one of these experiments on a goat, the accessory nerve of Willis was cut where it quits the vertebral canal, and the animal lost its voice immediately after the operation. Velpeau and Schellhammer are quoted in support of Arnold, who, from anatomical researches, experiments on animals, and pathological observations, concludes that the eleventh is the vocal nerve.

IX. *What is the influence of the pneumogastric nerve over the transformation of black into red blood?* The comb of all the fowls in which this nerve was divided sensibly lost its redness, and became blue and blackish. On post-mortem examination the lungs were observed redder than natural; the arteries, veins, and the heart were filled with a blackish blood, generally coagulated. The same results were observed in the rabbits. Probably this may be explained by the diminution in the number of the respirations. Legallois explained it on the supposition of occlusion of the glottis, but in some of the fowls and in young mammiferæ the glottis was never closed.

X. *What is the influence of the pneumogastric nerve over the movements of the heart?* The ancient physiologists attributed to this nerve a great influence over the movements of the heart. Bichat, Emmert, Legallois, and others, have demonstrated the fallacy of this opinion. Arnold observed that the movements were greatly accelerated, and very strong after the operation, and that they did not cease for some time after death, probably causing the accumulation of blood in the organ. Cooper found the pulsations of the heart feeble, but accelerated.

XI. *What is the change of temperature in animals after section of the pneumogastric nerve?* We find from the experiments of Arnold that the temperature diminished from one to two degrees (Reaumur) immediately, or at the latest an hour, after the section; that this diminution in the proper temperature of fowls and pigeons is from two to four degrees in the first twenty-four or forty-eight hours: the temperature then became so elevated as to reach, or sometimes to surpass the normal temperature by half a degree; in some cases the animal became cool shortly before death, while in others there was no elevation, the decrease continuing regularly from the moment of operation to death. A singular coincidence is thus remarked between the elevation and depression of the temperature, and the increase and diminution in the respirations. Thus it is clearly demonstrated by experiment that the pneumogastric nerve does not exercise a direct influence on the production of heat, but an indirect influence owing to the manner in which it affects the frequency of respiration.

XII. *When and in what manner does death occur after the function of the pneumogastric nerve is destroyed?* We have seen that the transformation of black into red blood is very imperfect: that the lungs, the heart, the great vessels, both veins and arteries, are filled with blood often coagulated, and therefore conclude that the cause of death is to be found on the one hand in the stagnation of blood in the lungs and heart disturbing their functions, and on the other to the arrested transformation of the blood. The animals then die by suffocation, and this suffocation is slow, if no obstacle prevents the air entering the lungs, but it is rapid if the glottis is closed, or if liquids or blood are effused into the trachea.

*Archives Générales de Médecine. Août*, 1840.

*On the Origin and Mode of Development of Zoospermata.* By M. Lallemand.

The zoospermata secreted by the testicles are susceptible, as all the products of secretion, of modification by all severe diseases, and by any cause greatly or protractedly disturbing the economy. Thus they diminish in number, in volume, in density, in vitality, according to the severity of the affection, and resist more or less putrid decomposition. They may become very scarce, and even be replaced by pyriform, ovoid, or spherical bodies. They are very mobile in their living state, and remarkable after death by their brilliant aspect, and the regularity of their forms. At the age of puberty these globules precede the appearance of the complete zoospermata, and replace them in old age and in certain pathological states, by which it appears that they are incomplete unformed secretions. The same phenomena are observed in healthy animals at each periodical return of heat. The zoospermata are most numerous in the secretory canals of the testicles, where they are almost dry, and heaped one upon another, most frequently in groups, of which all the heads are directed towards the epididymis, and the tails towards the surface of the testicle. In the vasa deferentia they separate and become more mobile and perfect. The fluids furnished by this canal, by the vesiculæ seminales, the prostate, and Cowper's glands have no other function than to favour their dilution and movements. The most perfect are always found the nearest to the excretory orifice. The most mobile, those which live the longest, are those which are voided during copulation. The ovules, secreted by the ovaries, are also according to M. Lallemand, living bodies before fecundation; they attain perfection in the oviducts after their separation from the ovary, and comport themselves exactly as the zoospermata.

[From the last proposition the editor of l'Expérience dissents, stating that the zoospermata are complete beings endowed with a peculiar independent life: but the ovules, on the contrary, are fragments of the ovary, which only participate in the general life of the individual, as all other parts of the organism, and do not acquire the independent life of spermatic animalcules until after fecundation.]

*L'Expérience.* 12 *Novembre,* 1840.

---

*On the Use of Chromic Acid in Microscopic Investigations.*
By Dr. Adolph Hannover.

After trying a variety of means the author found none at all to be compared with chromic acid for preserving not only the external form, but also the minute structure of the organized tissues, and for giving them a degree of hardness, such that the finest sections could be cut from them without disarranging their elementary parts. Even the different shades of colour (of the brain and spinal cord for example) were retained for many months; and the yellow colour which was imparted to transparent and very delicate objects was found to be even advantageous.

Dr. Hannover has prepared in this fluid cellular tissue of all kinds, elastic tissue, nuclei, the different kinds of epithelium, blood-globules, muscular fibres, cartilage and bone with their primary cells, nervous fibres from the nerves themselves as well as from the brain and spinal cord, ganglion-globules, eyes, and various other organs and tissues; and he has thus preserved them to all appearance unchanged, (with the exception of the yellow colour that some acquire,) and, by their hardening, admirably adapted for microscopic dissection and display.

To show how little influence the chromic acid has upon such vital actions, as may be observed after apparent death, he mentions that he has seen the ciliary movements continue for hours on the lining of the cerebral ventricles immersed in it, and has watched the repeated contractions of muscular fibres. As a proof also of the accuracy with which observations may be made on objects thus prepared, he describes many appearances confirmatory of the examinations which others have made of the fresh substances.

The mode of using this substance is, in ordinary cases, to mix one part of acid with 16 or 20 of water; but either stronger or weaker solutions may be safely used. If the substance is to be hardened, it must lie either for a short time in a strong solution, or for a longer time in a weak one; but the latter plan is preferable, because by the former the outer layers are hardened so soon that the access of the acid to the interior is hindered. For the same reason, to harden brain, it is necessary to divide it into small pieces. Blood-globules generally require a weak solution; muscle, bone, and cartilage, and most nerves will do in almost any.

*Müller's Archiv.* 1840, *p.* 549.

---

### *On Ciliary Motion.* By S. PAPPENHEIM.

MAYER of Bonn has, as is well known, given a description of the ciliary motion in the pericardium of the frog, and it has been confirmed by Valentin. I have completely and easily succeeded in observing the fact in tritons recently killed, and in seeing both the ciliæ and the mode in which they are fixed. I have also found them on the mucous membrane on the inner surface of the membrana tympani of frogs that had been dead 24 hours. In the pericardium of the triton, which is composed of parallel tendinous fibres, the ciliæ are placed upon the epithelium, which is transparent, apparently structureless, and containing a great number of very delicately-walled spherical cells. Each of these is surmounted by a diadem of ciliæ. Similar cells are found in the ciliary epithelium of the frog's tympanum, and in that of the pericardium also.

*Müller's Archiv.* 1840, *p.* 533.

---

### *On the Action of the Oblique Muscles of the Eye.* By A. W. VOLKMANN.

[WE have separated this portion from the paper on the motor influences of the cerebral and cervical nerves, because it is not immediately connected with the subject there specially treated of, and that it may the more certainly attract the attention of the many who, since the operation for strabismus has been practised, have been engaged in experiments on the muscles of the eyeball. As far as we have seen, none of these observers have detected the mode of action ascribed to the oblique muscles by Professor Hueck and the author of this paper, and now, we believe, generally admitted in Germany. At the same time none of the experiments lately performed are in any important degree opposed to the conclusions arrived at by these authors, and marked to all appearance with every necessary evidence of truth.]

In an admirable but little known treatise by Hueck of Dorpat, (on the rotation of the eye on its axis, Dorpat 1838,) it is shown that the office of the oblique muscles is to turn the eye upon its axis, so as to render it possible for the rays of light from any object to fall on identical spots on the retina, even when the head is inclined downwards; a condition on which, as is well known, singleness of vision depends. The observations (Nos. 2, 3, 4, 5,) related in the preceding paper fully confirm this view. In the dog, calf, and other animals experimented on, the axis round which the eye rotates is the visual axis itself, or at least its position is so little different from that of the latter, that when the oblique muscles act alone no motion of the pupil is discernible. In man a rotatory motion must also be produced by the action of the oblique; for since the muscles wind on the eyeball in the form of a semi-circle, rotation is unavoidable. But in man the axis of this rotation is not coincident with the axis of vision. While in the calf the direction in which the oblique muscles act intersects the visual axis at a right angle,—in man the intersection is at an acute angle. The inferior oblique arising from the lacrymal bone goes outwards and backwards, comes in contact with the eyeball at a point directly beneath the axis of rotation, and is then prolonged backwards and outwards, and after describing a semi-circle, is attached to the outer and posterior part of the eyeball. The rotation which it produces will therefore have an axis, which may be considered as passing from the outermost part of the iris to the point of insertion of the optic nerve; in short, an axis which, instead of coinciding with the visual axis, cuts it, and passes across it from the front and externally backwards and inwards. The necessary consequence is that when the inferior oblique acts *alone,* the pupil describes a portion of an arc of a circle; and, supposing that the view just advanced is correct, it must turn round the outermost point of the iris (which remains unmoved,) and be thus carried in a curved line upwards and outwards.

Albinus held this opinion respecting the action of the inferior oblique, but Bell and, with him, many modern authors have thought differently. The inferior oblique they think must move the eyeball so as to carry the pupil upwards and inwards, in the direction in which it is carried in wincing, and in which it is fixed during sleep. These motions, when automatic, are ascribed by Bell to the inferior oblique, and not to the combined actions of the superior and internal recti, which preside over voluntary motions exclusively. But the proofs for this opinion are insufficient. Bell divided the superior rectus in a rabbit, and found that the pupil was still moved upwards when the eye was irritated; but this might have been effected by the upper fibres of the retractor bulbi, (as in cap. 6.) He divided also the oblique muscles in a monkey, and it remained capable of all the voluntary motions of the eye, but the pupil on that side on which the inferior oblique was divided was *scarcely distinctly* moved upwards in wincing. But a slight motion must require an organ for its performance not less than a great one does; and Bell has therefore unintentionally proved that the motion of the pupil in wincing may depend on the recti. But, in fact, this motion is never produced by the inferior oblique; for, 1st, that muscle could only move the pupil upwards and *outwards;* 2d, its action must be connected with the axis-rotation of the eye of which no trace can be discerned in the act of wincing; 3d, the experiments already detailed prove that contractions of the inferior oblique are not connected with the twitching upwards of the pupil; 4th, that same twitching upwards continues after division of the inferior oblique. In short the position of the inferior oblique sufficiently indicates its action; it carries the pupil in the arc of a circle outwards and upwards.

In cases in which this rotation of the eye on its axis really takes place, it may be discerned in men as well as in animals, by a mode that Hueck pointed out. By fixing on a small vessel in the white of the eye, which (if possible) is directed transversely and horizontally across it, and then moving the head towards the shoulder, the vessel will be found not to alter its position; and it is therefore evident that the eye turns round in the direction opposite to that in which the head is inclined downwards.

The oblique muscles of the human eye, if they acted alone, would alter the position of the pupil and consequently the visual axis; but they do not act alone. The straight muscles determine the direction of the visual axis, and this being once fixed, the action of the oblique muscles can be no other than that of turning the eyeball round its unchanging axis.

*Müller's Archiv,* 1840. *Heft* iv. *p.* 480.

---

*On the Vis Nervosa of Haller.* By Dr. Marshall Hall.

The object of the present letter (the first of a series which, we presume, the author intends to publish in Müller's Archiv,) is to prove that what Haller called *vis nervosa,* Müller *vis motoria,* and Flourens *excitabilité,* is a power resident, not only in the nerves called motor, but in a whole system including centripetal and centrifugal nerves and the true spinal marrow; and that it is capable of acting along the spinal cord not only downwards but upwards. This doctrine is supported by precisely the same arguments as those which Dr. M. H. has elsewhere adduced in its behalf; and none of these meet the objection we have more than once brought against it, that there is *no proof whatever* that *a motor influence* ever travels along the *incident* nerves. All we know of the matter is, that the incident or afferent nerves, when excited at their peripheral extremity, produce a change in the central ganglion (the true spinal cord of vertebrata), by which a motor impulse is propagated along the efferent nerves; and, for anything that we know, or that can be proved (in our present state of ignorance as to the real mode by which these impressions are transmitted) to the contrary, the nature of the changes taking place in the incident and motor nerves may be as different as the direction of their propagation.

*Müller's Archiv.* 1840, *p.* 45.

